# Effects of Different Dietary Zinc (Zn) Sources on Growth Performance, Zn Metabolism, and Intestinal Health of Grass Carp

**DOI:** 10.3390/antiox12091664

**Published:** 2023-08-23

**Authors:** Yi-Chuang Xu, Hua Zheng, Jia-Cheng Guo, Xiao-Ying Tan, Tao Zhao, Yu-Feng Song, Xiao-Lei Wei, Zhi Luo

**Affiliations:** 1 Hubei Hongshan Laboratory, Fishery College, Huazhong Agricultural University, Wuhan 430070, China; xuyichuang@webmail.hzau.edu.cn (Y.-C.X.); zhenghua1@webmail.hzau.edu.cn (H.Z.); jiachengguo@webmail.hzau.edu.cn (J.-C.G.); txy7933@mail.hzau.edu.cn (X.-Y.T.); zhaotao2017@webmail.hzau.edu.cn (T.Z.); syf880310@mail.hzau.edu.cn (Y.-F.S.); xiaolei1205@webmail.hzau.edu.cn (X.-L.W.); 2 Laboratory for Marine Fisheries Science and Food Production Processes, Qingdao National Laboratory for Marine Science and Technology, Qingdao 266237, China

**Keywords:** dietary Zn sources, growth, Zn metabolism, intestinal health, grass carp

## Abstract

This research was conducted to investigate the effects of four dietary zinc (Zn) sources on growth performance, Zn metabolism, antioxidant capacity, endoplasmic reticulum (ER) stress, and tight junctions in the intestine of grass carp *Ctenopharyngodon idella*. Four Zn sources consisted of Zn dioxide nanoparticles (ZnO NPs), Zn sulfate heptahydrate (ZnSO_4_·7H_2_O), Zn lactate (Zn-Lac), and Zn glycine chelate (Zn-Gly), respectively. Grass carp with an initial body weight of 3.54 g/fish were fed one of four experimental diets for 8 weeks. Compared to inorganic Zn (ZnSO_4_·7H_2_O), grass carp fed the ZnO NPs and Zn-Gly diets exhibited better growth performance. Furthermore, grass carp fed the organic Zn (Zn-Lac and Zn-Gly) diets displayed enhanced Zn transport activity, improved intestinal histology, and increased intestinal tight junction-related genes expression compared to other groups. In comparison to other Zn sources, dietary ZnO NPs caused increased Zn deposition and damaged antioxidation capacity by suppressing antioxidant enzymatic activities and related gene expression in the intestine. Grass cap fed the ZnO NPs diet also exhibited lower mRNA abundance of endoplasmic reticulum (ER) stress- and tight junction-associated genes. According to the above findings, it can be concluded that dietary organic Zn addition (Zn-Lac and Zn-Gly) is more beneficial for intestinal health in grass carp compared to inorganic and nanoform Zn sources. These findings provide valuable insights into the application of organic Zn sources, specifically Zn-Lac and Zn-Gly, in the diets for grass carp and potentially for other fish species.

## 1. Introduction

Zinc (Zn) is a vital micro-element and plays a crucial role in multiple biological processes in vertebrates—including fish—such as growth, development, antioxidant, and metabolism [1,2,3,4]. The primary source of Zn acquisition for fish in natural conditions is the diet [5,6]. However, Zn is typically deficient in many plants used as alternative protein sources in the feeds [7]. Thus, dietary Zn supplementation is necessary. Zn sulphate (ZnSO_4_) is traditionally used in fish feeds, but it suffers from low bioavailability and poor absorption efficiency [3,8]. Studies demonstrated that dietary organic Zn source (2-hydroxy-4-(methylthio) butanoic Zn) or nano-Zn addition promotes growth performance and intestinal Zn accumulation of fish compared to the inorganic Zn source (ZnSO_4_) [9,10]. Therefore, compared with inorganic Zn sources, organic and nanoform Zn have higher bioavailability, making them effective alternatives to inorganic Zn in aquafeeds [10,11]. However, there is a little research investigating the impacts of different dietary Zn sources on Zn metabolism and intestinal health in fish.

The intestine is an essential organ responsible for digesting and absorbing nutrients from feed, and also for regulating the absorption and excretion of Zn in fish [5]. Zn transporters, ZnTs and ZIPs, work together with the metal response element binding transcription factor 1 (MTF1) to maintain cellular and systemic Zn homeostasis [2]. ZnT transporters are primarily responsible for moving Zn out of the cytosols and into either intracellular compartments or the extracellular space, while ZIP transporters play opposite roles in Zn transport compared to ZnT transporters [2]. It has been reported that ZnSO_4_ led to an increased expression of ZnT family-related genes but a decreased mRNA level of ZIP family-related genes in the intestine of yellow catfish [5]. ZnO NPs exhibited a similar pattern with increased expression of ZnT family-related genes and a reduced mRNA content of ZIP family-related genes in the liver of yellow catfish [6]. Recent research also revealed that methionine-chelated Zn (Zn-Met), compared to inorganic Zn source (ZnSO_4_), elevated the hepatic mRNA abundance of ZIP family-related genes of yellow catfish [11]. However, how dietary Zn sources differentially influence these Zn transporters’ expression in fish is lacking in fish.

The intestine is highly susceptible to damage, and its healthy status adversely impacts nutrients digestion and absorption. Studies have revealed that the histological features, antioxidant capacity, ER stress, and tight junctions serve as critical indicators of the intestinal health of fish [5,6,12,13]. Studies have been performed to investigate the influences of diets with Zn sources on these parameters in fish [5,6,7,14]. Previous research pointed out that the dietary organic Zn source (Zn-Lac) improved intestinal histology and increased mRNA abundance of genes associated with the antioxidant and tight junctions [14]. Furthermore, Meiler and Kumar [7] revealed that rainbow trout fed the amino acid-chelated Zn diet exhibited higher hepatic expression of antioxidant-related genes than those fed the ZnSO_4_ diet [7]. Chen et al. [5] showed that dietary ZnO NPs supplement inhibited antioxidant capacity and induced oxidative stress in the liver of yellow catfish. It also has been demonstrated that the hepatic activities of antioxidant-related enzymes were lower for Nile tilapia fed the ZnSO_4_ diet compared to those fed the ZnO NPs diet [15]. However, studies were still lacking about the different effects of dietary Zn sources on these parameters related to intestinal health. 

Grass carp *Ctenopharyngodon idella*, the highest-producing farmed fish in the world, recorded a production of 5.76 million tons in 2021 [16]. As an economically important species in China and several countries, grass carp production continues to increase annually. Though some studies have been conducted to compare the different impacts of dietary Zn sources (ZnO, ZnSO_4_, and ZnO NPs) on the growth of grass carp, there is still a lack of research evaluating the roles of organic, inorganic, and nanoform Zn sources in the growth and intestinal health [17,18]. Given the significant role of Zn in growth and physiology in fish and the higher bioavailability of organic and nanoform Zn compared to inorganic Zn sources, we hypothesize that dietary Zn sources significantly affected growth performance, Zn metabolism, and intestinal health in grass carp. Therefore, the current study was conducted to compare the impacts of four dietary Zn sources on growth, Zn metabolism, antioxidant capacity, ER stress, and tight junctions in the intestinal tissues of grass carp. The findings from this study will offer valuable insights into the choice for the optimal dietary Zn sources in the diets for grass carp and probably for other fish species.

## 2. Materials and Methods

### 2.1. Ethic Statement

This study on grass carp culture and management adhered to the Management Rule of Laboratory Animals (Chinese Order No. 676 of the State Council, revised 1 March 2017). All necessary measures were taken to minimize animal suffering. The Huazhong Agricultural University (HZAU) Ethics Committee approved the research protocols (identification code: Fish-2021-1031). 

### 2.2. Feed Formulation, Animals Feeding and Sampling

The feed formulation was prepared based on our recent research [6]. A previous study has established the Zn requirement of grass carp to be a 55.1 mg kg^−1^ diet, and four experimental diets were produced according to this requirement value [19], which contained ZnO nanoparticles (ZnO NPs, <50 nm, >97% in purity, #677450, Sigma-Aldrich, St. Louis, MO, USA), Zn sulfate heptahydrate (ZnSO_4_·7H_2_O, ≥99.5% in purity, #10024018, Sinopharm chemical reagent Co. Ltd., Shanghai, China), Zn lactate (Zn-Lac, ≥98% in purity, #S11095, Shanghai Yuanye Bio-Technology Co., Ltd., Shanghai, China), and Zn glycine chelate (Zn-Gly, ≥99% in purity, #S20212, Shanghai YuanYe Bio-Technology Co., Ltd.) (Supplementary Materials Table S1). In brief, all of the dry feed ingredients were ground, weighed, and thoroughly blended. The pre-weighed fish oil and soybean oil were added to homogenous dry ingredients and mixed thoroughly. Four different Zn sources were dissolved in the pre-weighed distilled water, respectively, and then mixed well with the corresponding feed ingredients to form a dough. Subsequently, the dough was passed through a 2-mm diameter die. Finally, the diets were dried using a dryer and stored in a freezer at 4 °C until used. Final dietary Zn contents were measured, and the contents were 81.12, 81.09, 81.38, and 81.56 mg kg^−1^ Zn for the ZnO NPs, ZnSO_4_·7H_2_O, Zn-Lac, and Zn-Gly diets, respectively. 

The culture and management of grass carp were conducted according to our previous publications [5,6]. In brief, a total of 240 uniformly sized grass carp (initial mean body weight: 3.54 ± 0.01 g, means ± S.D.) were randomly distributed among twelve 300-L circular aquariums, with 20 fish per tank. Each experimental diet was assigned to three replicate tanks, and the fish were fed to satiation twice daily for 8 weeks. The water temperature, dissolved oxygen, NH_4_-N, nitrite, and Zn levels were regularly monitored throughout the feeding experiment, with the corresponding values of 28.7–29.3 °C, ≥5.8 mg L^−1^, <0.09 mg L^−1^, <0.01 mg L^−1^, and 0.04 ± 0.00 mg L^−1^, respectively.

At the end of the 8-week feeding experiment, grass carp were subjected to a 24 h fasting before sampling to minimize prandial impacts. The fish were then euthanized using MS-222 (100 mg L^−1^, #E10521, Sigma-Aldrich, St. Louis, MO, USA). The final total weight of grass carp from each tank was determined. Twelve fish were randomly chosen from each tank. Six of these fish were used to measure their body length and body weight and dissected on ice to measure the weight of visceral and intestinal tissue mass, which were used to calculate the condition factor (CF) and viscerosomatic index (VSI). Intestine tissues from three fish per tank were sampled and fixed in 4% buffered formalin for histological analysis. Other samples were quickly frozen in liquid nitrogen and stored at −80 °C for the analysis of Zn contents, indices of oxidative stress, mRNA levels, and protein expression. The following formulas were used:

WG (weight gain, %) = 100 × (FBW − IBW).(1)

SGR (specific growth rate, %/d) = 100 × (ln (FBW) − ln (IBW))/day.(2)

FI (feed intake, g/fish) = the sum of feed consumed by fish throughout the experiment/fish number per tank.(3)

FCR (feed conversion rate) = dry feed fed (g)/wet weight gain (g).(4)

CF (condition factor) = 100 × (body weight, g)/(body length, cm) 3.(5)

VAI (visceral adipose index, %) = 100 × (visceral adipose weight, g)/(body weight, g).(6)

VSI (viscerosomatic index, %) = 100 × (viscera weight, g)/(body weight, g).(7)

### 2.3. Sample Analysis

#### 2.3.1. Hematoxylin and Eosin (H&E) Staining

For histological assessment, intestine samples were fixed in 4% buffered formalin overnight. Subsequently, they were dehydrated in graded ethanol concentrations and embedded in the paraffin. The samples were then sectioned at a thickness of 5 µm and stained with H&E, and eventually observed under a light microscope. General morphological analysis was conducted using Image J software (version 1.50i, NIH, Bethesda, MD, USA). Villi height, width, and area were measured and calculated based on the methods described in Eiras et al. [20].

#### 2.3.2. Proximate Analysis of Nutrient Components and Zn Contents

The moisture, ash, crude lipid, and crude protein levels of the diet were analyzed following the AOAC standard methods [21]. In brief, moisture content was determined through drying the sample at 105 °C. Ash content was analyzed by incinerating the diets in a muffle furnace at 550 °C. The Soxhlet ether extraction method was used to determine the lipid content, while the Kjeldahl method was used to measure the crude protein content via concentrated H_2_SO_4_ digestion. To determine the Zn contents in the diets, water, and tissues, inductively coupled plasma optical emission spectrometry (ICP-OES) was employed [22].

#### 2.3.3. Real-Time Quantitative PCR (qRT-PCR) Analysis

The qRT-PCR assay was conducted to analyze the gene abundances following our recent studies [6]. Total RNA was extracted using Trizol (#9108, TaKaRa, Tokyo, Japan). The integrity of the total RNA was measured using agarose gel electrophoresis (Supplementary Materials Figure S1). The purity of the total RNA was determined using a Nanodrop ND-2000 spectrophotometer (Thermo Fisher Scientific, Waltham, MA, USA) by measuring OD_260_, OD_280_, and OD_230_ (OD_260_/_280_ ≥ 1.8, OD_260_/_230_ ≥ 1.5) (Supplementary Materials Table S2), Total RNA was treated with DNase (#D7076, Beyotime Biotechnology, Shanghai, China) and then reverse transcribed into cDNA using a reverse transcription kit (#RR036, TaKaRa). qRT-PCR was conducted in a 25 μL reaction system which contained qPCR Mix (#RR430, TaKaRa), diluted cDNA template, forward and reverse primers, and double distilled H_2_O. In addition, no reverse transcriptase (NRT) and no-template control (NTC) analysis was performed. Gene-specific primers are provided in Supplementary Materials Table S3. We normalized the relative mRNA abundance values to the housekeeping genes (*β-actin* and *gapdh*), and calculated fold changes using 2^−ΔΔCt^ method.

#### 2.3.4. Immunoblot

To measure protein levels, immunoblot analysis was conducted based on a previously published protocol [6]. The samples were lysed using RIPA buffer (#P0013B, Beyotime Biotechnology, Shanghai, China). An equal amount of protein (20 mg) was loaded onto an SDS-polyacrylamide gel and transferred to the polyvinylidene fluoride (PVDF) membrane. The membranes were blocked with 4% bovine serum albumin (BSA, #ST023, Beyotime Biotechnology), followed by overnight incubation with primary antibodies at 4 °C. Afterwards, the membranes were incubated with appropriate secondary antibodies. After washing, the membranes were visualized using ECL (#P0018FS, Beyotime Biotechnology). The antibodies contained anti-NRF2 (1:1000, #16396-1-AP, Proteintech, Wuhan, China), anti-GAPDH (1:10000, #10494-1-AP, Proteintech, Chicago, IL, USA), anti-KEAP1 (1:1000, #80744-1-RR, Proteintech), and HRP-conjugated anti-rabbit IgG antibody (#7074, Cell Signaling Technology, Danvers, MA, USA).

#### 2.3.5. Measurement of Antioxidant Capacity

The activities of antioxidant enzymes, including superoxide dismutase (SOD, #S0101S), catalase (CAT, #S0056), glutathione peroxidase (GPx, #S0051), as well as the levels of glutathione (GSH, #S0053) and malondialdehyde (MDA, #S0131S) were determined via the corresponding commercial kits (Beyotime Biotechnology). The coefficient of variation was provided in Supplementary Materials Table S4.

### 2.4. Statistic Analysis

The statistical analysis was performed using SPSS 27.0 software (Armonk, NY, USA). The quantitative results are presented as means ± standard deviation (S.D.). The Shapiro–Wilk test and the Bartlett test were applied to evaluate data distribution normality and homogeneity of variances among groups, respectively. To determine significant differences among the treatments, a one-factor ANOVA and a post-hoc Duncan’s multiple range test were conducted. Statistical significance was set at *p* < 0.05. The principal component analysis (PCA) was conducted using chiplot (https://www.chiplot.online/, accessed on 6 August 2023). PCA was performed on the correlation matrix of values of growth performance, histological assessment, antioxidant capacities, and mRNA abundance of genes related to Zn transporters, ER stress, and tight junctions in the intestine of grass carp.

## 3. Results

### 3.1. Growth Performance

The impacts of different dietary Zn sources on the growth performance of grass carp are presented in Table 1. Among four groups, FBW and WG were higher for grass carp in the ZnO NPs and Zn-Gly groups than those in the ZnSO_4_·7H_2_O group (by 32.80–41.82% and 10.38 -13.61%, respectively, *p* < 0.001 and < 0.001, respectively). The ZnO NPs, Zn-Gly, and Zn-Lac group exhibited higher SGR than the ZnSO_4_·7H_2_O group (by 5.44–18.20%, *p* < 0.001). Grass carp fed the ZnO NPs diet demonstrated the higher FI than the other three groups (by 19.4–33.94%, *p* < 0.001). FCR was lower for grass carp fed the Zn-Lac diet than those in the ZnSO_4_·7H_2_O and Zn-Gly groups (by 7.0–7.93%, *p* = 0.066). Grass carp fed the ZnO NPs diet had higher CF than those fed the Zn-Gly diet (by 5.28%, *p* = 0.047). There were no significant differences in VAI, VSI, and survival among the four groups. Therefore, the nano-particle ZnO sources are better for promoting growth performance than the other three Zn sources for the grass carp.

Abbreviations: CF, condition factor; FBW, final mean body weight; FCR, feed conversion rate; FI, feed intake; HSI, hepatosomatic index; IBW, initial mean body weight; SGR, specific growth rate; VAI, visceral adipose index. VSI, viscerosomatic index; WG, weight gain.

WG (%) = 100 × (FBW − IBW).

SGR (%/d) = 100 × (ln (FBW) − ln (IBW))/day.

FI (g/fish) = the sum of feed consumed by fish throughout the experiment/fish number per tank.

FCR = dry food fed (g)/wet weight gain (g).

CF = 100 × (body weight, g)/(body length, cm)^3^.

VAI (%) = 100 × (visceral adipose weight, g)/(body weight, g).

VSI (%) = 100 × (viscera weight, g)/(body weight, g).

### 3.2. Intestinal Zn Level and mRNA Abundance of Zn Transporters

**Figure 1:**
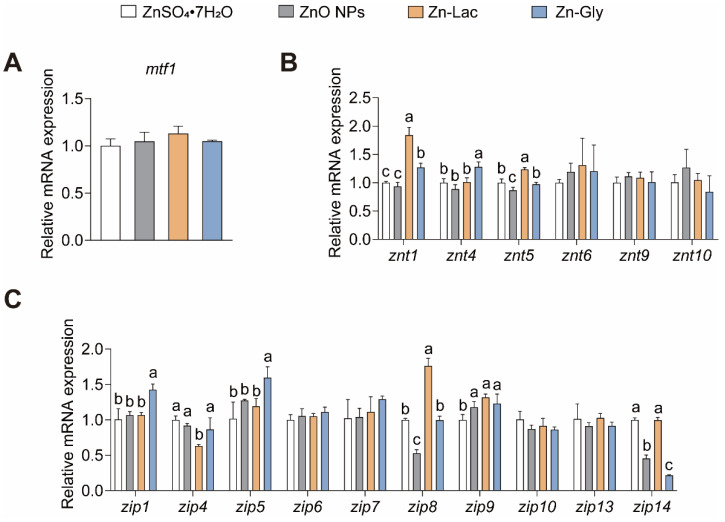
Different dietary Zn sources affected intestinal Zn transport-related genes expression in juvenile grass carp. (**A**) The mtf1 mRNA levels. MTF1, metal response element binding transcription factor 1; (**B**) The mRNA levels of ZnT family proteins; (**C**) The mRNA levels of ZIP family proteins. Values are shown as mean ± S.D. (*n* = 3 replicate tanks). Values with different letters (a–c) within the same row are significantly different at *p* < 0.05.

We then investigated the influences of different dietary Zn sources on the intestinal Zn level and gene expression of Zn transporters (Table 2 and Figure 1). The intestinal Zn level was higher for grass carp fed the ZnO NPs diet than those in the other three groups (by 38.24–80.13%, *p* = 0.003) (Table 2). There were no remarkable differences in mRNA abundance of *mtf1*, *znt6*, *znt9*, *znt10*, *zip6*, *zip7*, *zip10*, *zip13*, and *zip14* among four groups (Figure 1). The znt1 mRNA level was higher for grass carp in the Zn-Lac and Zn-Gly groups than those in the ZnO NPs and ZnSO_4_·7H_2_O groups (by 27.00–97.19%, *p* = 0.003). The mRNA level of *znt4* was higher in the Zn-Gly group than in the other three groups (by 26.94–44.38%, *p* = 0.002). The *znt5* mRNA expression was higher for grass carp in the Zn-Lac group than the other three groups (by 23.40–42.57%, *p* < 0.001). The mRNA levels of *zip1* and *zip5* were higher for grass carp in the Zn-Gly group than those in the other three dietary groups (by 33.69–41.85% and 25.08–56.93%, respectively, *p* = 0.002 and 0.009, respectively). The *zip4* expression was lower for grass carp in the Zn-Lac group than those in the other three groups (by 27.02–36.83%, *p* = 0.005). The *zip8* mRNA level was lower for grass carp fed the ZnO NPs diet than those in the other three groups (by 46.97–70.00%, *p* < 0.001). The *zip9* mRNA expression was lower for grass carp fed the ZnSO_4_·7H_2_O diet than those in the other three groups (by 15.20–24.02%, *p* = 0.016). The mRNA abundance of *zip14* was higher for grass carp fed the ZnSO_4_·7H_2_O and Zn-Lac diets than those fed the ZnO NPs and Zn-Gly diet (by 1.19- to 3.58-fold, *p* < 0.001).

### 3.3. Histological Assessment of Intestine

**Figure 2:**
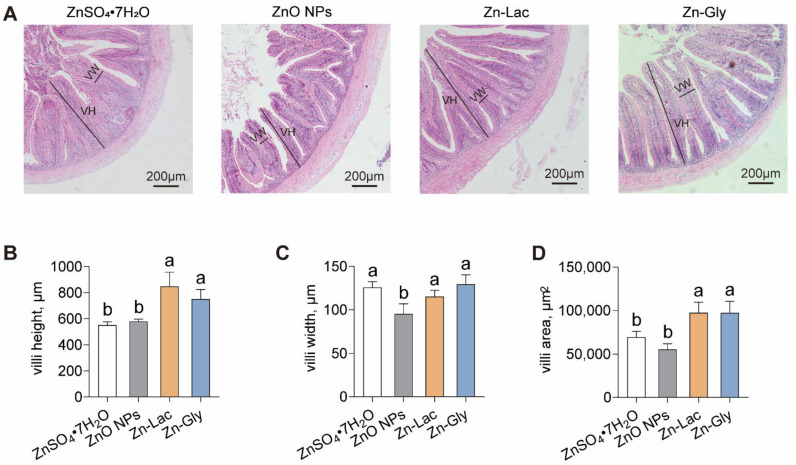
Different dietary Zn sources affected the intestinal histology of grass carp. (**A**) Representative H&E staining images. VH, villi height; VW, villi width. Bars represent 200 μm. (**B**–**D**) Statistical analysis of villi height, villi width, and villi area, related to Figure 2A. Values are shown as mean ± S.D. (*n* = 3 replicate tanks). Values with different letters (a,b) within the same row are significantly different at *p* < 0.05.

The intestinal morphology of grass carp was analyzed after feeding diets containing different Zn sources (Figure 2A). Unlike the other three dietary groups, the ZnO NPs diet damaged the histological structures of villi in grass carp (Figure 2A). The villi height and area were higher for grass carp fed two organic Zn sources (Zn-Lac and Zn-Gly) compared to those fed diets containing ZnO NPs and ZnSO_4_·7H_2_O (by 29.28–53.92% and 40.23–76.41%, respectively, *p* = 0.002 and 0.002, respectively) (Figure 2A–C). The villi width was lower for grass carp fed the ZnO NPs diet than those in the other three groups (by 17.25–26.40%, *p* = 0.008) (Figure 2A–C). These results demonstrate that two organic Zn sources contribute to better intestinal histology for the grass carp.

### 3.4. Indices of Antioxidant Capacity

**Figure 3:**
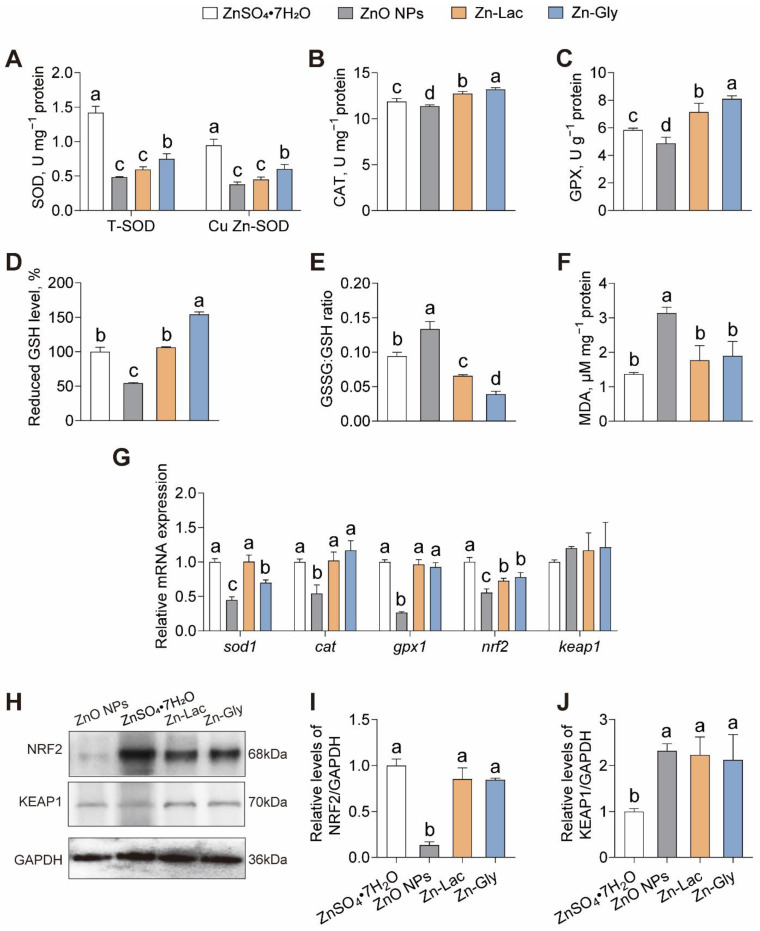
Different dietary Zn sources affected the intestinal antioxidant capacity of grass carp. (**A**) superoxide dismutase (SOD) activity. (**B**) catalase (CAT) activity. (**C**) glutathione peroxidase (GPX) activity. (**D**) reduced glutathione level. (**E**) oxidized/reduced glutathione ratio. (**F**) malondialdehyde. (MDA) content. (**G**) mRNA levels of oxidative stress-related genes. (**H**–**J**) Western blot and statistical analysis of NRF2 and KEAP1 expression. Values are shown as mean ± S.D. (*n* = 3 replicate tanks). Values with different letters (a–d) within the same sub-figure are significantly different at *p* < 0.05.

To explore the roles of different Zn sources in antioxidant capacity, we evaluated the indices of antioxidant capacity (Figure 3). The activities of total SOD (T-SOD) and Cu Zn-SOD were higher for grass carp fed the ZnSO_4_·7H_2_O diet than those in the other groups (by 0.90- to 1.93-fold and 0.56- to 1.50-fold, respectively, *p* < 0.001 and < 0.001, respectively) (Figure 3A). The CAT and GPX activities, as well as reduced GSH level, displayed similar trends among four groups, with higher levels observed in the Zn-Gly group than those in the other three groups (by 3.66–16.13%, 13.48–66.42%, and 18.34–54.21%, respectively, *p* < 0.001, <0.001, and <0.001, respectively) (Figure 3B–D). The GSSG: GSH ratio was higher in the ZnO NPs group than those in the other three groups (by 0.42- to 2.42-fold, *p* < 0.001) (Figure 3E). The MDA content was significantly higher for grass carp fed the ZnO NPs diet than those in the other three groups (0.66- to 1.29-fold, *p* < 0.001) (Figure 3F).

Next, the intestinal expressions of genes and proteins related to antioxidant capacity were analyzed. As shown in Figure 3G, the mRNA level of *sod1* was higher in the ZnSO_4_·7H_2_O and Zn-Lac groups than those in the Zn-Gly and ZnO NPs groups (by 0.43- to 1.22-fold, *p* < 0.001). The mRNA abundance of *cat*, *gpx1*, and *nrf2* was lower for grass carp in the ZnO NPs group than those in the other three groups (by 45.90–53.63%, 71.34–73.48%, and 23.69–44.62%, respectively, *p* < 0.001, <0.001, and <0.001, respectively) (Figure 3G). The protein level of NRF2 was lower for grass carp fed the ZnO NPs diet than the other groups (by 84.18–86.63%, *p* < 0.001), while no significant differences were observed among the other three groups (Figure 3H,I). Conversely, the protein expression of KEAP1 was lower for grass carp in the ZnSO_4_·7H_2_O group than those in the other three groups (by 52.87–56.91%, *p* = 0.005) (Figure 3H,I). Thus, our findings indicate that grass carp fed the ZnO NPs diet exhibited the weakest antioxidant capacity among the four groups.

### 3.5. mRNA Expression of Gens Related to ER Stress

**Figure 4:**
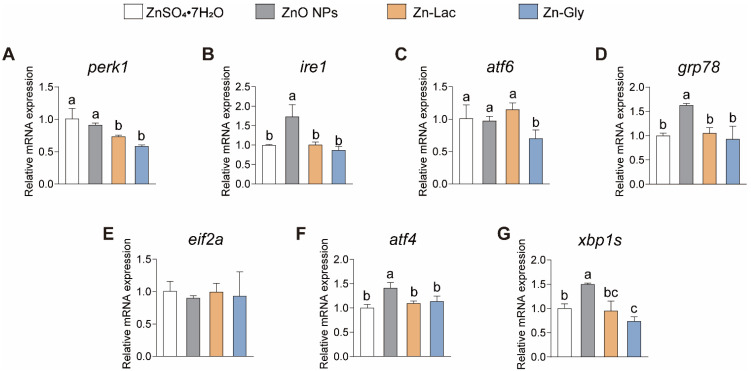
Different dietary Zn sources affected the endoplasmic reticulum stress-related genes expression in the intestinal of grass carp. (**A**) *perk*. (**B**) *ire1*. (**C**) *atf6*. (**D**) *grp78*. (**E**) *eif2a*. (**F**) *atf4*. (**G**) *xbp1s*. ATF4, activating transcription factor 4; ATF6, activating transcription factor 6; EIF2a, eukaryotic translation initiation factor 2α; GRP78, glucose-regulated protein 78; IRE1, inositol-requiring enzyme 1; PERK, Eukaryotic translation initiation factor 2-alpha kinase 3; XBP1, X-box binding protein 1. Values are shown as means ± S.D. (*n* = 3 replicate tanks). Values with different letters (a–c) within the same gene are significantly different at *p* < 0.05.

Considering the essential pathophysiological role that ER stress plays in vertebrates [23,24], we then investigated the impacts of different dietary Zn sources on the intestinal mRNA levels of genes related to ER stress in grass carp (Figure 4). Our findings indicated the expression of *ire1*, *grp78*, *atf4*, and *xbp1s* showed similar trends among four groups, with higher levels observed in the ZnO NPs group than the other three groups (by 71.69–98.37%, 54.91–75.19%, 23.99–40.99%, and 0.50- to 1.03-fold, respectively, *p* < 0.001, =0.001, =0.003, and =0.032, respectively) (Figure 4). The mRNA level of *perk* was lower for grass carp fed the two organic Zn (Zn-Lac and Zn-Gly) diets compared to those fed the ZnO NPs and ZnSO_4_·7H_2_O diets (by 19.53–41.98%, *p* = 0.001) (Figure 4A). Grass carp fed the Zn-Gly diet showed lower mRNA abundance of *atf6* than the other three groups (by 28.08–39.02%, *p* = 0.022) (Figure 4C). Taken together, the above results suggest that ZnO NPs diet is more likely to induce ER stress in the intestine compared to other dietary Zn sources.

### 3.6. mRNA Expression of Genes Related to Tight Junctions

**Figure 5:**
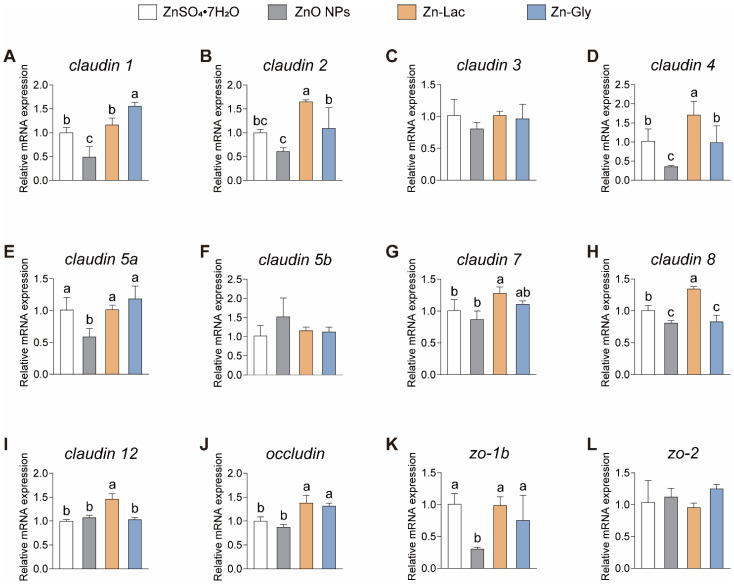
Different dietary Zn sources affected the mRNA abundance of tight junction-related genes in the intestinal of grass carp. (**A**) *claudin 1*. (**B**) *claudin 2*. (**C**) *claudin 3*. (**D**) *claudin 4*. (**E**) *claudin 5a*. (**F**) *claudin 5b*. (**G**) *claudin 7*. (**H**) *claudin 8*. (**I**) *claudin 12*. (**J**) *occluding*. (**K**) *zo-1b*. (**L**) *zo-2*. Values are shown as mean ± S.D. (*n* = 3 replicate tanks). Values with different letters (a–c) within the same gene are significantly different at *p* < 0.05.

A tight junction is considered as an indicator of intestinal healthy status in fish [25,26]. In this study, the intestinal mRNA expression of genes related to tight junctions was analyzed in grass carp fed with different dietary Zn sources (Figure 5). The mRNA levels of *claudin 1*, *claudin 5a*, and *zo-1b* were lower for grass carp in the ZnO NPs group than those in the other three groups (by 51.01–68.47%, 41.42–50.00%, and 59.38–69.54%, respectively, *p* < 0.001, =0.009, and =0.016, respectively) (Figure 5). Grass carp fed the Zn-Lac diet showed higher mRNA abundance of *claudin 2*, *4*, *8*, and *12* than those in the other three groups (by 0.51- to 1.70-fold, 0.67- to 3.78-fold, 34.27–66.19%, and 36.13–46.12%, respectively, *p* = 0.003, =0.006, <0.001, and <0.001, respectively) (Figure 5). Furthermore, the mRNA level of *claudin 2* and *4* was higher in the Zn-Gly group than in the ZnO NPs group (by 78.62% and 1.75-fold, respectively, *p* = 0.003 and 0.006, respectively), while there is no marked difference in *claudin 2* and *4* mRNA levels between the ZnSO_4_·7H_2_O and Zn-Gly groups (Figure 5). The mRNA expression of *claudin 8* was higher for grass carp in the ZnSO_4_·7H_2_O group than in the ZnO NPs and Zn-Gly groups (by 20.60–23.77%, *p* < 0.001), but no significant difference was observed in *claudin 8* mRNA abundance between ZnO NPs and Zn-Gly groups (Figure 5H). Additionally, grass carp fed the two organic Zn (Zn-Lac and Zn-Gly) diets showed higher *occludin* expression in comparison with those fed the ZnO NPs and ZnSO_4_·7H_2_O diets (by 31.70–58.17%, *p* < 0.001) (Figure 5J). In general, these results indicate that the Zn-Lac diet is more beneficial for intestinal tight junctions in grass carp compared to other dietary Zn sources.

### 3.7. The Principal Component Analysis (PCA)

As shown in Supplementary Materials Figure S2, the PCA was performed on the whole set of average values with 49.63% in the first principal component (PC1) and 19.13% in the second (PC2). The first two principal components (PCs) explained 68.76% of the total variability. The PCA results revealed significant differences among the ZnSO_4_·7H_2_O group, ZnO NPs group, and the two groups of organic Zn sources (Zn-Lac and Zn-Gly) (Supplementary Materials Figure S2). Similarities were observed between the Zn-Lac group and the Zn-Gly group (Supplementary Materials Figure S2). Analyzed samples of the same cultivar grouped together demonstrated that ZnSO_4_·7H_2_O, Zn-Lac, and Zn-Gly groups were richest in the protein level of NRF2 and the mRNA abundance of *claudin4*, which were negatively regulated by the ZnO NPs source (Supplementary Materials Figure S2, Table S5). In addition, our results showed that the activity of T-SOD was more sensitive to grass carp fed the ZnSO_4_·7H_2_O diet than to those fed the Zn-La and Zn-Gly diets (Supplementary Materials Figure S2, Table S5).

## 4. Discussion

In the current study, we first explored the impacts of four dietary Zn sources on the growth of grass carp. Results showed that grass carp fed the ZnO NPs and Zn-Gly diets exhibited higher growth performance, compared to those fed the ZnSO_4_·7H_2_O diet, while grass carp fed the Zn-Lac diet displayed the lowest FCR among four Zn sources diets, demonstrating that nanoform and organic Zn sources were utilized better than inorganic Zn sources. Similarly, Faiz et al. [17] disclosed the enhanced growth in grass carp fed the ZnO NPs diet compared to those fed inorganic Zn diets. Meanwhile, Mohseni et al. [27] reported that beluga sturgeons fed an organic Zn (Zn-methionine) diet exhibited higher growth performance and lower FCR than those fed an inorganic Zn diet. In our study, the ZnO NPs group had the highest FI, but FCR was not significantly different from the ZnSO_4_·7H_2_O and Zn-Gly groups, suggesting that the superior growth performance of grass carp in the ZnO NPs group was primarily attributed to increased food intake. The CF is critical for assessing the health status of fish [7]. We found that grass carp fed the Zn-Gly diet displayed lower CF, whereas those fed the ZnO NPs diet showed higher CF. Previous studies on rainbow trout revealed that CF was not significantly affected by the dietary Zn level of rainbow trout [7].

The intestine is the primary organ for absorbing mineral elements derived from diet. It was demonstrated that, compared to the inorganic Zn source (ZnSO_4_), the dietary organic Zn source (2-hydroxy-4-(methylthio) butanoic Zn) and nano Zn promoted more Zn accumulation in the intestine of fish [9,10]. Zn transport is mainly regulated by ZnTs, ZIPs, and MTF1 [2]. This study discovered that ZnO NPs are more easily bioaccumulated in the intestine, while organic Zn sources have greater influences on the regulation of Zn transporter-related gene (*znt1*, *znt4*, *znt5*, *zip1*, *zip4*, *zip5*, *zip8*, and *zip14*) expression. Cellular Zn efflux is primarily mediated by ZnT1 [28,29]. ZnT4 and ZnT5, located in the cytoplasmic membrane, control Zn efflux from cytoplasm to maintain cytosolic Zn homeostasis [2]. On the contrary, ZIP1, ZIP4, ZIP5, ZIP8, and ZIP14 are located at the plasma membrane and mainly function as importers of Zn from the extracellular space [2,30]. Based on our findings, it appears that, in comparison to inorganic and nanoform Zn, the intake of organic Zn in grass carp enhances the mRNA levels of the ZIP family-related genes to facilitate Zn entry into the cell. However, excess Zn needs to be excreted by up-regulating the mRNA abundance of the ZnT family-related genes to counteract the increasing intestinal Zn contents and contribute to maintaining Zn homeostasis. Chen et al. [5] also demonstrated that Zn overload caused the up-regulation of ZnT5 and 7 expressions in the intestine, which helps to delay intracellular Zn toxicity. Similarly, it was found that diets with organic Mn sources (Mn glycine chelate and Mn-MHA) showed higher mRNA expression of both Mn absorption and efflux-related genes in the intestine of yellow catfish compared to MnO_2_ NPs and diets with inorganic Mn sources (MnSO_4_ and MnO_2_), indicating that dietary organic mineral sources have a greater impact on the activity of mineral transport [26]. To our best knowledge, studies were very scarce about the impact of organic Zn sources on Zn transport-related gene expression. Here, we report for the first time that organic Zn is more likely to induce alterations in Zn transport-related gene expression than inorganic and nanoform Zn. In addition, our research demonstrated that nanoform Zn exerts a relatively slight effect on the mRNA abundance of Zn transport-related genes among the four Zn sources. However, it is important to note that dietary nanoform Zn results in more Zn accumulation in the intestine. Studies have demonstrated that nanoparticles can enter the cell through endocytosis [31,32]. Chen et al. [6] also revealed that dietary ZnO NPs up-regulated mRNA abundance of genes associated with endocytosis, which indicates that, besides Zn transporters, there are alternative pathways in which ZnO NPs enter the cell.

Intestinal histology is a crucial physiological indicator for assessing the health status of the intestine [13,26]. This study exhibited that grass carp fed diets containing organic Zn sources exhibited greater intestinal villi height and areas in comparison to those fed the ZnO NPs and ZnSO_4_·7H_2_O diets. The height and area of villi are closely associated with the absorption area of the intestine [20]. Therefore, our results indicate that organic Zn diets promote an increase in the intestinal absorption area compared to nanoform and inorganic Zn diets. Similarly, Xu et al. [26] demonstrated that the villi height was higher for fish fed the organic Mn source diets in comparison to those for fish fed the nanoform and inorganic Mn source diets. It was revealed that intestinal histology is closely influenced by tight junctions [33]. The tight junction, located in the most apical region of the cell, plays important roles in ions and solutes diffusion, barrier function, and cell proliferation [34]. This study demonstrated that grass carp fed the Zn-Lac diet showed the highest mRNA abundance of *claudin 2*, *4*, *7*, *8*, and *12*, while the Zn-Gly group showed the highest mRNA abundance of *claudin 1*. Conversely, the mRNA levels of *claudin 5a* and *zo-1b* were the lowest for grass carp fed the ZnO NPs diet. Furthermore, the ZnSO_4_·7H_2_O group displayed up-regulation of *claudin 1*, *4*, and *8* expressions compared to the ZnO NPs group. Transmembrane proteins, including claudins and occludin, along with adaptor proteins like ZO-1 and ZO-2, are crucial components of the tight junction [34]. The increase in mRNA abundance of the tight junction-related genes indicates that the organic Zn source diets promote the enhancement of the tight junction. Similarly, previous research reported that diets with organic Zn sources (Zn-Gly and Zn-Lac) up-regulate the mRNA level of *occludin* in the intestine compared to the ZnSO_4_ diet [35]. Taken together, our findings suggest that organic Zn sources promote the enhancement of the tight junction compared to inorganic and nanoform Zn sources.

The intestinal antioxidant capacity serves as a valuable index for evaluating the intestinal health in fish [5,26]. We revealed that grass carp fed the inorganic and organic Zn diets elevated intestinal antioxidant-related enzyme (T-SOD, Cu Zn-SOD, CAT, and GPX) activities and reduced-GSH content, and there was a lower GSSG: GSH ratio and lower MDA levels, compared to those fed the ZnO NPs diet. SOD, CAT, and GPx are the essential enzymes involved in the antioxidant process. SOD is responsible for catalyzing the dismutation of superoxide radicals to H_2_O_2_, which is then metabolized to H_2_O and O_2_ by CAT and GPx [6,36]. GPx reduces H_2_O_2_ to H_2_O with the electrons coming from GSH [6,36]. MDA, an end product of lipid peroxidation, is commonly used as an indicator of oxidative stress [6]. Thus, the up-regulation of their activities and down-regulation of the GSSG: GSH ratio and MDA content displayed the improvement of antioxidant capacity for grass carp fed the inorganic and organic Zn diets in comparison to those fed the ZnO NPs diet. However, Kishawy et al. [37] showed that the dietary ZnO NPs supplement, compared to ZnO and Zn-Met addition, up-regulated SOD activity and down-regulated MDA content in the muscles of tilapia. It was also demonstrated that tilapia fed the ZnO NPs diet exhibited higher hepatic SOD and CAT activities and lower MDA content than those fed the organic Zn source (Zn acetate) diet [38]. Therefore, the effects of Zn sources on the antioxidant capacity of fish are tissue- and fish species-dependent. We also revealed that grass carp in inorganic and organic Zn groups tend to have higher intestinal mRNA abundance of *sod1*, *cat1*, and *gpx1* than those in the ZnO NPs group. The effects of different Zn sources on antioxidant-related gene expression and enzymatic activities were found to be similar, which further supported that dietary inorganic and organic Zn sources, in comparison with ZnO NPs, enhanced the intestinal antioxidant capacity of fish. In addition, we demonstrated that the mRNA and protein expressions of NRF2 were higher for grass carp fed the inorganic and organic Zn sources diets than those fed the ZnO NPs diet. Conversely, the mRNA and protein abundance of KEAP1 was the lowest for grass carp in the ZnSO_4_·7H_2_O group. NRF2 is a vital transcription factor involved in positively controlling the expression of antioxidant-related genes, while KEAP1 acts as an inhibitor of NRF2 [39]. Thus, the increase in NRF2 level and the decrease in KEAP1 abundance promote the expression of antioxidant-related genes, leading to an enhanced antioxidant capacity [39]. Taken together, the above findings suggest that the ZnO NPs diet resulted in oxidative stress while the inorganic and organic Zn sources diets contributed to the enhancement of antioxidant capacity.

ER is a crucial organelle for maturing secreted and transmembrane proteins, while various external factors and intrinsic cellular events can interfere with the protein-folding capacity of the ER, resulting in a situation identified as ER stress [23]. Our findings indicated that, in the ZnO NPs group, there was a higher mRNA abundance of *ire1*, *grp78*, *atf4*, and *xbp1s*. Grass carp fed the two organic Zn diets displayed a lower mRNA level of *perk* and grass carp fed the Zn-Gly diet exhibited the lowest mRNA abundance of *atf6*. ATF6, IRE1, and PERK serve as sensors of ER stress, while GRP78 has a higher affinity for binding misfolded or unfolded proteins under ER stress [40]. The IRE1-XBP1 and PERK-eIF2α-ATF4 represent the most evolutionarily conserved arm of the ER stress [23]. Thus, the ZnO NPs diet tends to induce ER stress in the intestine compared to other dietary Zn sources, whereas diets with inorganic and organic Zn sources inhibited ER stress. Similarly, Xu et al. [26] found that compared with dietary organic Mn sources and MnSO_4_ supplement, the dietary addition of MnO_2_ NPs inhibited intestinal *grp78* and *atf4* expression of yellow catfish, which provides evidence that dietary nano-form mineral sources contribute to the induction of ER stress. Studies investigating the distinct impacts of dietary Zn sources on ER stress are currently limited. For the first time, this study elucidated the impact of different diets with Zn sources on ER stress. It is worth noting that oxidative stress and ER stress are tightly related. Oxidative stress can initiate ER stress, while an increase in the load of protein folding within the ER can lead to oxidative stress by elevating ROS content [41,42]. Thus, the ability of diets with inorganic and organic Zn sources in alleviating ER stress compared to diets with ZnO NPs may be attributed to their antioxidant effects.

## 5. Conclusions

In this study, we discovered that (1) ZnO NPs and Zn-Gly diets facilitated growth more than the inorganic Zn source for the grass carp; (2) organic Zn sources exhibited greater Zn transport activity, while ZnO NPs were more readily bioaccumulated in the intestine; (3) organic Zn sources improved intestinal histology in grass carp; (4) the ZnO NPs diet caused oxidative and ER stress, while the Zn-Lac diet benefited the integrity of a tight junction in the intestine of grass carp. Our research elucidated the influences of different dietary Zn sources on growth performance, Zn metabolism, antioxidant capacity, endoplasmic reticulum stress, and tight junctions in intestinal tissues of grass carp, which provides valuable insights for applying organic Zn sources in the diets for grass carp and probably for other fish species.

## Figures and Tables

**Table 1:** Influences of dietary Zn sources on growth performance and feed utilization of juvenile grass carp.

	ZnSO_4_·7H_2_O	ZnO NPs	Zn-Lac	Zn-Gly
IBW, g/fish	3.54 ± 0.01	3.54 ± 0.01	3.55 ± 0.01	3.54 ± 0.01
FBW, g/fish	16.81 ± 0.37 ^c^	22.32 ± 1.58 ^a^	18.33 ± 0.06 ^bc^	18.55 ± 0.47 ^b^
WG, %	374.83 ± 10.6 ^c^	531.59 ± 46.91 ^a^	416.75 ± 1.28 ^bc^	425.85 ± 14.11 ^b^
FI, g/fish	14.91 ± 0.93 ^b^	19.97 ± 1.57 ^a^	15.29 ± 0.43 ^b^	16.72 ± 1.01 ^b^
SGR, %/d	2.78 ± 0.04 ^c^	3.29 ± 0.14 ^a^	2.93 ± 0.00 ^b^	2.96 ± 0.05 ^b^
FCR	1.12 ± 0.05 ^a^	1.06 ± 0.05 ^ab^	1.03 ± 0.03 ^b^	1.11 ± 0.02 ^a^
CF	1.81 ± 0.15 ^ab^	1.87 ± 0.08 ^a^	1.80 ± 0.07 ^ab^	1.78 ± 0.09 ^b^
VAI, %	1.09 ± 0.49	1.15 ± 0.38	1.21 ± 0.45	1.10 ± 0.38
VSI, %	8.46 ± 1.55	8.02 ± 1.37	8.06 ± 1.06	7.60 ± 1.13
Survival, %	96.67 ± 5.77	98.33 ± 2.89	96.67 ± 2.89	98.33 ± 2.89

Values are shown as means ± S.D. (*n* = 3 replicate tanks. For IBW, FBW, WG, SGR, FCR, and survival, 18–20 fish each tank; for CF, VSI, and HSI, 6 fish each tank). Values with different letters (a–c) within the same row are significantly different at *p* < 0.05.

**Table 2:** Zn concentration (mg kg^−1^ live weight) in the intestine of juvenile grass carp at the end of the growth experiment.

	ZnSO_4_·7H_2_O	ZnO NPs	Zn-Lac	Zn-Gly
Zn	26.89 ± 5.72 ^b^	37.17 ± 3.98 ^a^	20.88 ± 3.33 ^b^	20.63 ± 2.47 ^b^

Values are shown as means ± S.D. (*n* = 3 replicate tanks). Values with different letters (a,b) within the same row are significantly different at *p* < 0.05.

## Data Availability

Data will be made available on request.

## Supplementary Materials

The following supporting information can be downloaded at: https://www.mdpi.com/article/10.3390/antiox12091664/s1, Table S1: Formulation and proximate composition of the experimental diets (% dry matter); Table S2: The quality of RNA samples; Table S3: Primers used for quantitative real-time PCR (qRT-PCR) analysis; Table S4: The coefficient of variation for the indices of antioxidant capacity; Table S5: The representative loading scores of the principal component analysis (PCA); Figure S1: The measurement of RNA integrity in the samples using RNA gel (n = 3 replicate tanks); Figure S2: The principal component analysis (PCA) shows the relationship of four dietary Zn sources.

## Abbreviations

ATF: activating transcription factor; CAT, catalase; CF, Condition factor; EIF2A, Eukaryotic translation initiation factor 2A; FAO, Food and Agriculture Organization of the United Nations; FBW, final mean body weight; FCR, feed conversion rate; GAPDH, glyceraldehyde-3-phosphate dehydrogenase; GPX, glutathione peroxidase; GRP78, Endoplasmic reticulum chaperone BiP; GST, glutathione S-transferase; H&E, Hematoxylin and eosin; IRE1, serine/threonine-protein kinase/endoribonuclease IRE1-like protein; KEAP1, kelch-like ECH-associated protein 1; MTF1, metal-regulatory transcription factor 1; NRF2, nuclear factor erythroid-specific related factor 2; PERK, Eukaryotic translation initiation factor 2-alpha kinase 3; qRT-PCR, Real-time quantitative PCR; SGR, specific growth rate; SOD, superoxide dismutase; VSI, viscerosomatic index; WG, weight gain; XBP, X-box binding protein; ZO, tight junction protein.
