# Supplementary material for: Effects of Different Dietary Zinc (Zn) Sources on Growth Performance, Zn Metabolism, and Intestinal Health of Grass Carp
Source: Antioxidants (Basel). 2023 Aug 23;12(9):1664. doi: 10.3390/antiox12091664 (PMC10525721; doi:10.3390/antiox12091664)
Supplement: Supplementary file 1 [file antioxidants-12-01664-s001.zip › antioxidants-2544160-supplementary.pdf]

Supplementary Materials

Effects of different dietary zinc (Zn) sources on growth performance, Zn metabolism, and intestinal health of grass carp

Yi-Chuang Xu[1], Hua Zheng[1], Jia-Cheng Guo[1], Xiao-Ying Tan[1], Tao Zhao[1], Yu-Feng Song[1], Xiao-Lei Wei[1], Zhi Luo[1,2,*]

[1] Hubei Hongshan Laboratory, Fishery College, Huazhong Agricultural University, Wuhan 430070, China

[2] Laboratory for Marine Fisheries Science and Food Production Processes, Qingdao National Laboratory for Marine Science and Technology, Qingdao 266237, China

*Corresponding author at: Zhi Luo, Hubei Hongshan Laboratory, Fishery College, Huazhong Agricultural University, Wuhan 430070, China, Tel.: +86-27-8728-2113; Fax: +86-27-8728-2114;

E-mail address: luozhi99@mail.hzau.edu.cn; luozhi99@aliyun.com (Z. Luo).

Table S1 Formulation and proximate composition of the experimental diets (% dry

matter)

Ingredients ($g\ kg^{-1}$)	$ZnSO_4\bullet 7H_2O$	ZnO NPs	Zn-Lac	Zn-Gly
Fish meal	80	80	80	80
casein	35	35	35	35
Rice bran	110	110	110	110
Soybean meal	200	200	200	200
Rapeseed meal	250	250	250	250
Wheat Starch	250	250	250	250
Fish oil	20	20	20	20
Soybean oil	10	10	10	10
Choline chloride	1.5	1.5	1.5	1.5
Vitamin premix	4	4	4	4
Mineral premix	7.5	7.5	7.5	7.5
DL-methionine	0.15	0.15	0.15	0.15
L-lysine	0.4	0.4	0.4	0.4
L-threonine	0.4	0.4	0.4	0.4
Lecithin	1	1	1	1
$Ca(H_2PO_4)_2\cdot H_2O$	20	20	20	20
Cellulose	9.9	10.008	9.923	9.939
$ZnSO_4\cdot 7H_2O$	0.150			
ZnO NPs		0.042		
Zn-Lac			0.127	
Zn-Gly				0.111
Proximate analysis %, dry weight				
Moisture	8.57	8.43	8.66	8.86
Crude protein	30.82	31.09	31.88	31.13
Crude lipid	6.31	6.63	6.58	6.49
Ash	7.67	7.54	7.76	7.59
Zn ($mg\ kg^{-1}$)	81.09	81.12	81.38	81.56

Vitamin premix (mg or IU per kg diet): niacin, 50; calcium pantothenate, 30; pyridoxine hydrochloride, 12; menadione nicotinamide bisulfite, 7; folic acid, 6; thiamine hydrochloride, 6; riboflavin, 3; D- biotin, 1; cyanocobalamine, 0.03. retinylacetate, 10000IU; cholecalciferol, 1000IU; all-rac-a-tocopheryl acetate, 30IU;

Mineral mixture (mg per kg diet): NaCl, 500; $FeSO_4\cdot 7H_2O$, 400; $MnSO_4\cdot H_2O$, 40; $CuSO_4\cdot 5H_2O$, 12; $CaIO_3\cdot 6H_2O$, 1.5; Na_2SeO_3, 0.45; $CoSO_4$, 1.

Table S2 The quality of RNA samples.

	Concentration (ng μL^{-1})	A260/280	A260/230
	702.54	2.03	2.21
$ZnSO_4$•$7H_2O$	884.05	2.00	2.29
	918.11	2.03	2.09
	662.38	1.97	2.29
ZnO NPs	584.77	1.95	2.34
	666.06	1.97	2.36
	797.19	2.01	2.34
Zn-Lac	804.34	1.99	2.33
	608.29	1.94	2.33
	553.56	1.97	2.22
Zn-Gly	754.23	1.95	2.37
	814.24	2.02	2.25

Table S3 Primers used for quantitative real-time PCR (qRT-PCR) analysis.

Genes	Forward primer (5'-3')	Reverse primer (5'-3')	Accession no.
atf4	CCTCTCCTCTTCCTAC GCTG	CGACATCCAATCCATG CCAG	AY437846
atf6	TGACACCTCTGTTCC TGACC	CCTCAGCAAAACCAC TCGAG	KT279356
β-actin	ACCCTGAAGTACCCC ATCGA	CAGAGGCATACAGGG ACAGC	DQ211096.1
cat	CCAGAGAGAGTTGT GCATGC	CTTCACCGCAAATCCT CGAG	FJ560431
claudin 1	TGGCTTCTCTGGGTC TCTTG	TGAACTTCCCCTGGTA GCTG	XM05188046 4
claudin 2	CAACATCGTGACAGC GGTAG	GCACAGGCCATGACA GAAAA	XM05187679 5
claudin 3	ATCGTGACGGCTCAG ATCAT	GGACGGTCAGAAGGA TGGAT	XM05186218 5
claudin 4	GGGTTTGTGGATGAG	CACACCTGAGACGAT	XM05187744

	CTGTG	GCATG	3
claudin 5a	TGGGTCTGATCCTATGCGTC	ATGGAGTCGTGGATCTTGCA	XM051904492
claudin 5b	ACGGGTCAGATGCAGTGTAA	CAGAGGCACCAACACAAACA	KU200696
claudin 7	GTGCCCTGATGATTGTAGCG	AAAGGGCTGCAACAATGAGG	KT445866
claudin 8	CCGGTCTGGGTCTACTGATC	CATCGATGAGCAAGGGGTTG	XM051863391
claudin 12	ATGACATCAAGAACTCGCGC	ACAGGTTATCGTAGCGCTCA	XM051864743
eif2a	GTTCAACAGAGTGCATGCCA	GACGCCTCTCTTCTCTTCGA	KJ126860
gapdh	GGGAAACTGTGGAGGGATGG	TGCAGCCTTGACCACTTTCT	GQ245759.1
gpx1	TCTGAAGTATGTCCGTCCGG	GTTCCAGGCGATGTCATTCC	JX854447
grp78	CGACGAGAAGAAGGAAAGCG	CTGGTTCTTCGCAGCATCTC	FJ436356
ire1	GCAAAGTCCCACACACCAAT	AGTAACTCCGTCATGCACCA	MG797683
keap1	ACGCAGGAGGAGTTCTTCAA	TAGGTGTGAGTGAGTGGCAG	XM051896876
mtf-1	GGCTCCTCCTCCTCCTACAA	CCGACTGGTATGGCTGTTGT	MK770661
nrf2	CTTGCAGATCCCGTTTACCG	TCTAGGCCCACGATGTTCTC	KY807076
occludin	CACCTTCATCGTTCTGCTCG	AACTGAACTGATCCGGACGT	KF193855

perk	CTGGATGGAAGAATCGCAGC	GCCTCCATACTCTCCCTGTC	KX906957
sod1	GTCCGCACTTCAACCCTTAC	TTCCTCATTGCCTCCCTTCC	GU901214
xbp1s	GCACAGACAGCAAGAGACAG	ACCAAACCCAAATCGCTCAC	KU509247
zip1	CTAGATGAGGGCTTCCCGCT	CATTCAACGAGTGCCGTCCA	OP106599
zip4	TCTTGCGGGCGTCTATTTCT	AAGTTATGGATGCCGTCGCC	OP106600
zip5	TTAGGTGATTTGGCGGTGCT	TGGAGCATTTCGGGCATCAT	OP106601
zip6	GAAGGTTTGTCCAGCGGTCT	CATACCCGCCTTCAGCAAGA	OP106602
zip7	CCAGTGCAGTCAAACACGGA	AGAGTGAGAGTGCGAGTGTC	OP106603
zip8	AAAACGGCTCTCTGTCCACG	ATTACCCAACCCAAAGGCGG	OP106604
zip9	TCCAGAAGCAGCAAGGACTG	AGCAGGAGCCTTATGCAACA	OP106605
zip10	TTCTGGTGCCCATCCTCAAC	CCTGCTAGTGCAGTCAGTCC	OP106606
zip13	ATCAGCCCTTACACAGAGCC	TACGAGTGGCTTTCCAACGG	OP106608
zip14	GCGCTATGCCAACTCCAATG	TAACATCCACCCGTTCCAGC	OP106609
znt1	GCAACCCGATGTGGTGATTG	GGCTGTTGTTGTGATTCCCG	OP106610
znt4	CTCAATCAGTCCGGG	CACACCCTCCAGCAG	OP106612

	CATCT	AATGA	
znt5	ATGTGCTGTCTGGTG GAGTG	TGATGAAGCGAGGCA GAGAC	OP106613
znt6	CGAGAGGAAAGTGT CGCCTT	ACACCAGATTCAACA CGCCA	OP106614
znt9	TTGGCGTGGGTTTAT ACGGG	GGCGATGTAACGCAT GTTGG	OP106617
znt10	ACTGGCTAAAGGAC GGAACG	CCACCAGGACAACAG GAGAG	OP106618
zo-1b	CCCAAGCACGACAA AGACAA	GTGGATTCGGCCCTTG ATTC	XM05188508 2
zo-2	GACGTCCATCATTGT GTCGG	TAATCACGGCTATCCA CGCT	XM05190425 2

Abbreviations: *atf*, activating transcription factor; *cat*, catalase; *eif2a*, Eukaryotic translation initiation factor 2A; *gapdh*, glyceraldehyde-3-phosphate dehydrogenase; gpx, glutathione peroxidase; *grp78*, Endoplasmic reticulum chaperone BiP; *ire1*, serine/threonine-protein kinase/endoribonuclease IRE1-like protein; *keap1*, kelch-like ECH-associated protein 1; *mtf1*, metal-regulatory transcription factor 1; *nrf2*, nuclear factor erythroid-specific related factor 2; *perk*, Eukaryotic translation initiation factor 2-alpha kinase 3; *sod*, superoxide dismutase; *xbp*, X-box binding protein; zo, tight junction protein.

Table S4 The coefficient of variation for the indices of antioxidant capacity.

	$ZnSO_4 \cdot 7H_2O$	ZnO NPs	Zn-Lac	Zn-Gly
T-SOD	6.41%	1.08%	6.74%	10.09%
Cu Zn-SOD	9.36%	9.26%	7.76%	10.60%
CAT	2.79%	1.33%	2.02%	1.44%
GPX	2.23%	9.17%	8.97%	2.64%
Reduced GSH	5.89%	8.25%	2.72%	11.05%

GSSG:GSH ratio	6.45%	1.59%	0.72%	2.29%
MDA	2.80%	5.31%	23.91%	22.23%

Table S5 The representative loading scores of the principal component analysis (PCA).

feature	PC1	PC2
Protein level of NRF2	-0.323	0.106
mRNA abundance of claudin4	-0.300	-0.075
Activity of T-SOD	-0.130	0.309

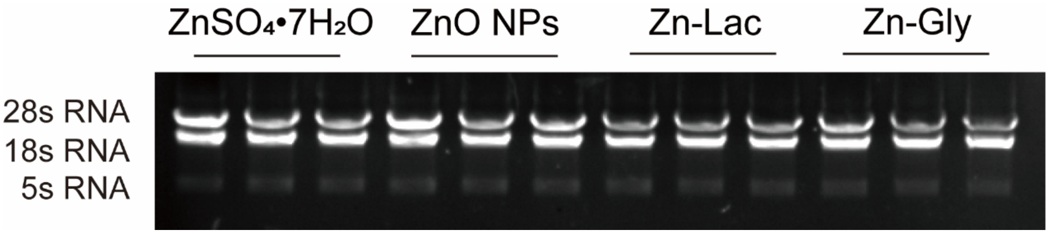

Figure S1. The measurement of RNA integrity in the samples using RNA gel (n=3 replicate tanks).

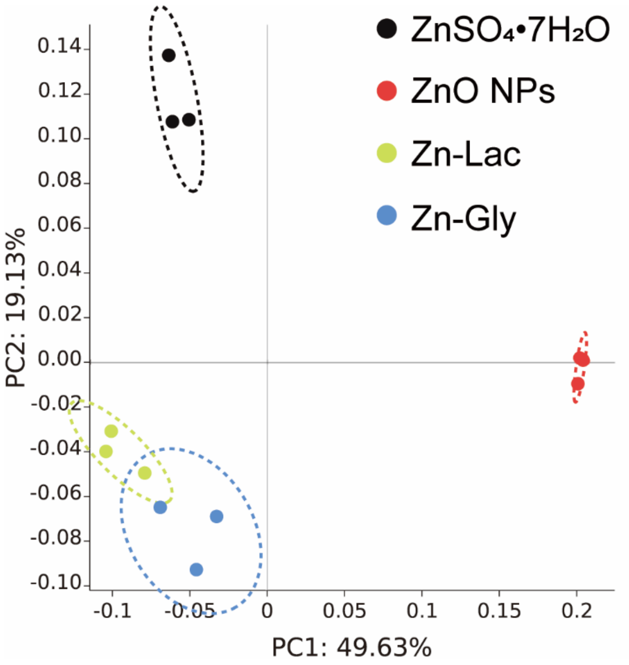

Figure S2. The principal component analysis (PCA) shows the relationship of four dietary Zn sources.
